# Interspecies differences in PTH-mediated PKA phosphorylation of the epithelial calcium channel TRPV5

**DOI:** 10.1007/s00424-017-1996-9

**Published:** 2017-05-22

**Authors:** Mark K van Goor, Sjoerd Verkaart, Teunis J van Dam, Martijn A Huynen, Jenny van der Wijst

**Affiliations:** 1 Department of Physiology, Radboud University Medical Center, PO Box 9101, 6500 HB Nijmegen, The Netherlands; 2 Centre for Molecular and Biomolecular Informatics, Radboud Institute for Molecular Life Sciences, Radboud University Medical Center, Nijmegen, The Netherlands; 3 Theoretical Biology and Bioinformatics, Department of Biology, Faculty of Science, Utrecht University, Utrecht, The Netherlands

**Keywords:** TRP channel, Calcium, Hormone signaling, PKA

## Abstract

The epithelial calcium (Ca^2+^) channel TRPV5 (transient receptor potential vanilloid 5) is expressed in the distal convoluted tubule of the kidney and facilitates active Ca^2+^ reabsorption. This process is instrumental for the maintenance of Ca^2+^ homeostasis. Therefore, all aspects of TRPV5 function are tightly regulated by the calciotropic parathyroid hormone (PTH). Rabbit (rb)TRPV5 channel activity was shown to be stimulated upon PTH-mediated protein kinase A (PKA) phosphorylation. Since there is incomplete conservation of the PKA consensus motif (RR/QxT) across species, the aim of this study was to extend these findings to humans and characterize the expression and function of human (h)TRPV5. Functional differences between rbTRPV5 and hTRPV5 upon PTH stimulation were investigated using ^45^Ca^2+^ uptake assays, Fura-2 Ca^2+^ imaging, and cell surface biotinylation. While PTH treatment enhanced rbTRPV5 channel activity, it did not stimulate hTRPV5 activity. Mutation of the human RQxT motif into rabbit RRxT (hTRPV5 Q706R) partially restored the sensitivity to PTH. An ancestral sequence reconstruction of TRPV5 orthologues demonstrated that the change in the RRxT motif coincides with the creation of another putative PKA motif (RGAS to RRAS) in the amino terminus of hTRPV5. Interestingly, a constitutively phosphorylated hTRPV5 mutant (hTRPV5 S141D) displayed significantly decreased channel function, while its plasma membrane abundance was increased. Taken together, PTH-mediated stimulation of TRPV5, via PKA, is not conserved in humans. Our data suggest that PTH regulation of TRPV5 is altered in humans, an important observation for future studies that may add to new concepts on the role of PTH in renal Ca^2+^ handling.

## Introduction

Of all the transient receptor potential (TRP) family members, TRPV5 and TRPV6 are the most calcium (Ca^2+^)-selective channels, and play a unique role in Ca^2+^ homeostasis by facilitating active Ca^2+^ (re)absorption in kidney and intestine [14]. Regulation of renal and intestinal Ca^2+^ transport processes, together with dynamic Ca^2+^ storage in bone, maintains plasma Ca^2+^ levels within a range of 2.2–2.7 mmol/L [15, 19, 33]. This intricate balance is essential for a myriad of physiological processes including muscle contraction, neuronal excitability, heart automaticity, and bone integrity [20, 21, 27].

Within the kidney, Ca^2+^ transport is extensively regulated via the combined efforts of the parathyroid hormone (PTH) and 1,25-dihydroxyvitamin D_3_ (1,25(OH)_2_D_3_) [18]. Under the governance of these calciotropic hormones, the kidney reabsorbs approximately 99% of all filtered Ca^2+^ via two spatially distinct transport processes. In the proximal tubule and thick ascending limb of Henle’s loop, a total of 85–90% of Ca^2+^ is reabsorbed in a passive paracellular manner, dependent on the electrochemical gradient from tubular lumen to interstitial fluid. Next, active transcellular Ca^2+^ reabsorption occurs through the epithelial cells of the distal convoluted tubule and connecting tubule. This highly regulated process determines the final urinary Ca^2+^ excretion, since downstream segments are unable to reabsorb Ca^2+^ [2, 9, 18]. TRPV5 mediates Ca^2+^ entry at the apical membrane of the distal convolution, and because of its unique location and activity, this channel is often called the gatekeeper of active Ca^2+^ reabsorption [12, 13]. Further evidence of this key role is provided by *Trpv5* knock-out mice which have a sixfold increase in urinary Ca^2+^ excretion, together with malformations in bone structure, intestinal hyperabsorption of Ca^2+^ and significantly higher levels of 1,25(OH)_2_D_3_ [16, 25].

In concordance with its function as gatekeeper, TRPV5 is extensively regulated at (i) the transcriptional level, (ii) via trafficking to and from the plasma membrane, and (iii) at the level of single channel activity. It is known that PTH receptor activation mediates the latter two processes through downstream signaling to PKA (protein kinase A) and PKC (protein kinase C), which phosphorylate various residues present in the intracellular termini of rabbit (rb)TRPV5 [3, 6]. One of the carboxy-terminal phosphorylation sites (T709), present as an RRxT consensus PKA motif, has been identified as the primary site of PTH-mediated PKA stimulation [6]. De Groot et al. showed that the TRPV5 T709D mutant channel, which mimics constitutive phosphorylation, has significantly decreased Ca^2+^-dependent inactivation and increased open probability at single channel level. In an earlier study, Nilius et al. also demonstrated a key role for the carboxy terminus in Ca^2+^-dependent channel inactivation of TRPV5, discovering that this mode of inhibition was reliant on an interaction of the TRPV5 carboxy terminus with the universal Ca^2+^-sensing protein calmodulin (CaM) [22, 23]. Indeed, two key CaM-binding sites (W702 and R706) were identified in the carboxy terminus of TRPV5. Mutation of these residues led to loss of CaM binding and resulted in reduced Ca^2+^-dependent channel inactivation of TRPV5 [5]. Interestingly, the TRPV5 T709D mutant also displayed reduced CaM binding. In contrast, no significant changes were found in CaM binding for the non-phosphorylated TRPV5 T709A mutant [5]. This suggests that PKA phosphorylation stimulates the TRPV5 channel by abrogating its interaction with CaM, thereby reducing Ca^2+^-dependent inactivation and increasing open probability. Even though there is limited conservation of the CaM and PKA recognition motifs among species (Fig. 3a), the non-transcriptional effect of PTH signaling has only been studied in rbTRPV5 [1, 3, 6, 11].

The aim of the present study was, therefore, to extend these findings to humans and characterize the expression and function of the human (h)TRPV5 channel. We investigated the functional implications of sequence differences between rbTRPV5 and hTRPV5 with respect to PTH signaling and CaM binding by using a combination of biochemical approaches.

## Methods

### Buffers

Lysis buffer: 50 mM Tris-HCl (pH 7.5), 150 mM NaCl, 1 mM EDTA, 1 mM EGTA, 1% (*v*/*v*) Triton X-100, 1 mM sodium orthovanadate, 10 mM sodium-glycerophosphate, 50 mM sodium fluoride, 10 mM sodium pyrophosphate, 270 mM sucrose, and the freshly added protease inhibitors pepstatin A (1 μg/ml), PMSF (1 mM), leupeptin (5 μg/ml), and aprotinin (1 μg/ml). Buffer A: 50 mM Tris-HCl (pH 7.5) and 0.1 mM EGTA. TBS-Tween (TBS-T): Tris-HCL (200 mM, pH 7.5), 150 mM NaCl, and 0.2% (*v*/*v*) Tween-20. SDS-PAGE sample buffer (5×): 10% (*w*/*v*) SDS, 25% (*v*/*v*) β-mercaptoethanol, 50% (*v*/*v*) glycerol, 300 mM Tris-HCl (pH 6.8), and 0.05% (*v*/*v*) bromophenol blue. ^45^Ca^2+^ uptake KHB buffer: 1 μCi/ml ^45^Ca^2+^, 110 mM NaCl, 5 mM KCl, 1.2 mM MgCl_2_, 0.1 mM CaCl_2_, 10 mM Na-acetate, 2 mM NaH_2_PO_4_, and 20 mM HEPES (pH 7.4, NaOH), with added inhibitors of voltage-gated Ca^2+^ channels (10 μM felodipine and 10 μM verapamil). ^45^Ca^2+^ uptake stop buffer: 110 mM NaCl, 5 mM KCl, 1.2 mM MgCl_2_, 10 mM Na-acetate, 0.5 mM CaCl_2_, 1.5 mM LaCl_2_, and 20 mM HEPES (pH 7.4, NaOH). Biotinylation wash buffer (PBS-CM): PBS, 1 mM MgCl_2_ and 0,5 mM CaCl_2_ (pH 8.0 NaOH). Biotinylation quenching buffer: PBS-CM, 0.1% (*w*/*v*) BSA (bovine albumin serum). Fura-2 imaging HEPES medium: 132.0 mM NaCl, 4.2 mM KCl, 1.4 mM CaCl2, 1.0 mM MgCl_2_, 5.5 mM D-glucose, 10 mM HEPES/Tris, and pH 7.4.

### Cell culture

HEK293 (human embryonic kidney 293) cells were grown in Dulbeccos Modified Eagles medium (DMEM, Bio Whittaker Europe) supplemented with 10% (*v*/*v*) fetal calf serum (PAA), 2 mM L-glutamine, and 10 μl/ml non-essential amino acids at 37 °C in a humidity-controlled incubator with 5% (*v*/*v*) CO_2_. For transient transfection, cells were seeded as 1.2–1.4 × 10^6^ cells per 6-well and transfected after 6 h with 1 μg of the respective DNA constructs using polyethyleneimine (PEI, Brunschwig Chemie) with a DNA:PEI ratio of 1:6. The cells were cultured for 48 additional hours and uniform expression was confirmed by their green fluorescence (at 488 nm) prior to the experiments.

### DNA constructs

Generation of the HA-tagged rabbit TRPV5 pCINeo-IRES-GFP construct was described previously [32]. The human TRPV5 DNA was a kind gift of Dr. Ji-Bin Peng and was cloned into the HA pCINeo-IRES-GFP vector via standard PCR cloning. Mutations in both TRPV5 plasmids were introduced using the QuikChange site-directed mutagenesis method (Stratagene), according to the manufacturer’s instructions. The hPTHR cDNA was obtained as described previously [5]. DNA sequencing validated the sequence of all constructs, and DNA purity was confirmed using the NanoDrop 2000c (Isogen Life Science).

### Cell surface biotinylation

HEK293 cells were seeded on poly-L-lysine-coated 6-well plates and transfected with the indicated constructs. To determine TRPV5 abundance at the plasma membrane, the cells were washed once with 2 ml ice-cold PBS-CM, and subsequently incubated with 0.5 mg/ml biotin (Ez-link Sulfo-NHS-LC-LC-biotin, Pierce Biotechnology) for 30 min at 4 °C. Following, the biotinylation reaction was stopped by washing the cells twice with quenching buffer and once with PBS. Finally, cells were lysed in 0.3 ml lysis buffer. Cell lysates were collected, centrifuged at 4 °C for 15 min at 16,000*g*, and protein concentration was determined using the Bradford method. Equal amounts of protein were incubated with neutravidin beads (Pierce Biotechnology) for 2 h at 4 °C. To retrieve biotin-tagged TRPV5 from the beads, cells were washed three times in lysis buffer and once in buffer A, and TRPV5 was eluted with sample buffer.

### Calmodulin binding assay

HEK293 cells were lysed in lysis buffer containing either 1 mM CaCl_2_ or 5 mM EGTA (negative control) and lysates were cleared by centrifugation at 4 °C for 15 min at 16,000*g*. After determination of the protein concentration by the Bradford method, 0.5–1 mg lysate was incubated with calmodulin (CaM)-coupled agarose beads (Sigma Aldrich) for 2 h at 4 °C under gentle agitation. Subsequently, the beads were washed three times with lysis buffer and once with Buffer A. Proteins were eluted in SDS sample buffer.

### Immunoblotting

Samples were subjected to electrophoresis on 8% (*w*/*v*) SDS-PAGE gels and then transferred to PVDF membranes. The membranes were blocked for 30 min at room temperature in TBS-T containing 5% (*w*/*v*) non-fat dry milk (NFDM). Subsequently, they were immunoblotted overnight at 4 °C with the respective primary antibody (in TBS-T containing 5% (*w*/*v*) NFDM). The HA antibody (1:5000) was obtained from Cell Signaling Technology, and the beta-actin (1:10,000) from Sigma (A5441). The next day, blots were washed with TBS-T to remove unbound primary antibody and then incubated with horseradish peroxidase (HRP) conjugated secondary goat anti-mouse antibody (1:10,000, Brunschwig Chemie) for 1 h at room temperature. After subsequent washes, the protein was visualized with chemiluminescence SuperSignal West reagent (Thermo Fisher Scientific), and imaged with the Biorad ChemiDoc XRS.

### ^45^Ca^2+^ uptake assay

One day after transfection, HEK293 cells expressing the respective TRPV5 proteins were re-seeded from 6-well into poly-L-lysine-coated 24-well plates. The next day, cells were pretreated for 30 min with 25 μM BAPTA-AM. Subsequently, they were washed once in KHB buffer and incubated for 10 min with ^45^Ca^2+^ (1 μCi/ml) in KHB buffer. The blocker ruthenium red (RR, 10 μM) was used as a control for TRPV5-mediated uptake. The assay was stopped by three sequential washes with ice-cold stop buffer, and the amount of ^45^Ca^2+^ uptake was measured with liquid scintillation counting.

### Intracellular Ca^2+^ measurements using fura-2-AM

HEK293 cells were seeded on fibronectin-coated coverslips (inner diameter, 25 mm) and transfected with the appropriate TRPV5 constructs. After 24 h, cells were loaded with 3 μm Fura-2-AM and 0.01% (*v*/*v*) Pluronic F-129 (both from Molecular Probes) in DMEM medium at 37 °C for 20 min. After loading, the cells were washed twice with PBS and allowed to equilibrate at 37 °C for another 10 min in HEPES medium. Next, the cells were placed on an inverted microscope using an incubation chamber containing HEPES medium, and intracellular Ca^2+^ levels were calculated from the fluorescence emission ratio of 340 and 380 nm excitation. Details on the microscopy procedures and quantitative image analysis have been described previously [7]. All measurements were performed at room temperature.

### Ancestral sequence reconstruction

Sequences of TRPV5/TRPV6 orthologous sequences were collected from EnsEMBL 83 (on January 7, 2016) protein family ENSFM00500000270358 [34]. To pinpoint the time of duplication a phylogenetic tree was constructed using RAxML (version 7.2.8a) [28] with the pre-aligned sequences from EnsEMBL using the following options: -f a-m PROTCATLG-x 134601139-N 100. Our analysis suggests that the TRPV5 specific marsupial duplication as shown by EnsEMBL may not be correct, but in fact comprise both TRPV5 and TRPV6 marsupial sequences. The common carboxy-terminal PKA motif RRNT (TRPV5) versus RRGT (TRPV6) in the marsupial sequences supports this. Hence, we put the origin of the TRPV5/TRPV6 duplication at the therian ancestor and not the eutherian ancestor as suggested by EnsEMBL. We have no evidence to support that the multiple Platypus TRPV5/TRPV6 orthologs may or may not similarly be mislocalized in the gene tree, however alternative localization of Platypus sequences will have no effect on ancestral sequence reconstruction. Ancestral sequences of the amino-terminal and carboxy-terminal PKA motifs were manually reconstructed based on parsimony and under the assumption that the gene tree topology is identical to the species tree as used in EnsEMBL 83, with the TRPV5/TRPV6 duplication positioned at the therian ancestor.

### Statistical analysis

All data are shown as mean +/− standard error of the mean (SEM). Statistical significance (*p* < 0.05) was determined by analysis of variance and a Dunnett post-hoc test.

## Results

### Human TRPV5 is insensitive to PTH-mediated signaling

**Fig. 1:**
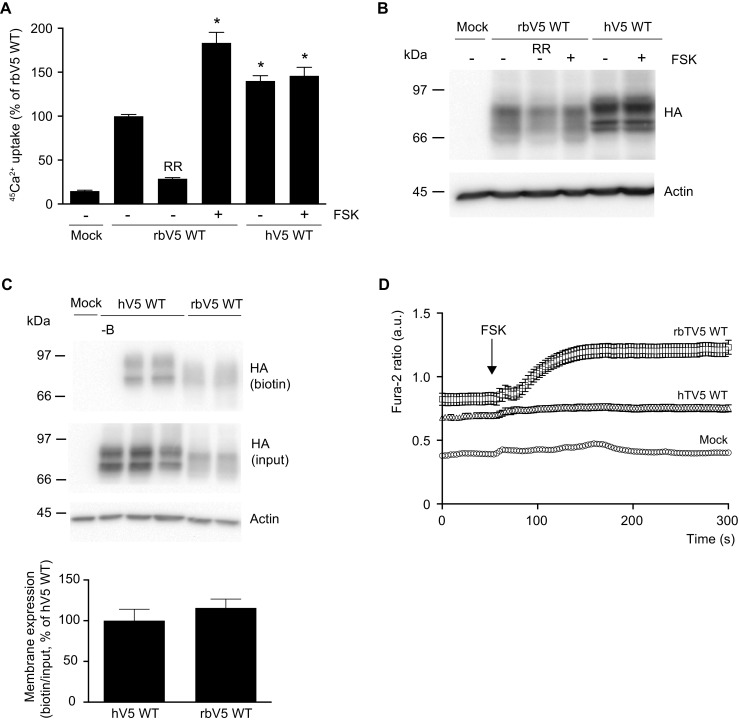
Expression and function of human TRPV5. **a** ^45^Ca^2+^ uptake assay of HEK293 cells transfected with either human (hV5 WT) or rabbit (rbV5 WT) wild type TRPV5. Forskolin (FSK, 10 μM) was added directly with ^45^Ca^2+^ at the start of the experiment. The uptake is depicted as percentage of rbWT in mean ± SEM (*N* = 9, from three independent experiments). *Asterisk* indicates *p* < 0.05 compared to rbWT. Ruthenium red (RR, 10 μM) is used to define TRPV5-mediated uptake. **b** Cell lysates of the respective Ca^2+^ uptake experiments were immunoblotted with HA antibody, using β-actin as loading control. A representative immunoblot is shown. **c** Cell surface biotinylation of HEK293 cells transfected with human (hV5 WT) or rabbit (rbV5 WT) wild type TRPV5. Samples were analyzed by immunoblotting with HA antibody. The biotin fraction represents the TRPV5 present at the plasma membrane (*top panel*), and input demonstrates TRPV5 expression in total cell lysates (*middle panel*), with β-actin as loading control. Representative immunoblot of three independent experiments is depicted. Control without added biotin is indicated as *-B*. The *bottom bar graph* depicts the quantified summary of the relative membrane expression compared to input, shown as percentage of hV5 WT. **d** Averaged Fura-2 ratio in arbitrary units (a.u.) of HEK293 cells expressing mock (*n* = 51), rabbit TRPV5 wild type (rbV5 WT; *n* = 69), and human TRPV5 wild type (hV5 WT; *n* = 101) upon forskolin (FSK) stimulation at *t* = 60 s indicated by the *arrow*. The total cell number is obtained in at least three independent experiments

**Fig. 2:**
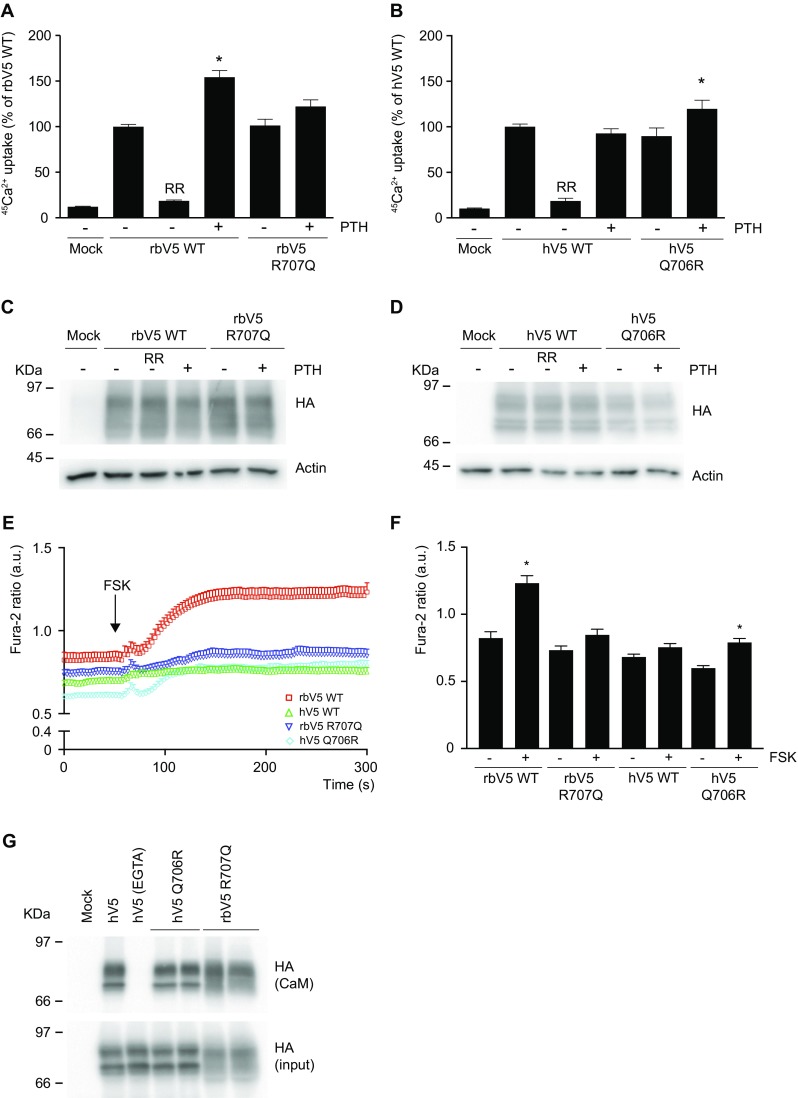
Importance of the RR/QxT motif. **a**, **b** ^45^Ca^2+^ uptake assay of HEK293 cells transfected with the PTH receptor PTH1R, and either rabbit wild type TRPV5 (rbV5 WT) or the R707Q mutant (**a**), and human wild type TRPV5 (hV5 WT) or the Q706R mutant (**b**). PTH (10 μM) was added directly with ^45^Ca^2+^ at the start of the experiment. Data is presented as percentage of rbV5 WT and hV5 WT respectively, in mean ± SEM (*n* = 9, from three independent experiments). *Asterisk* indicates *p* < 0.05 compared to rbV5 WT and hV5 WT respectively. Ruthenium red (RR) is used to define TRPV5-mediated uptake. **c**, **d** Cell lysates of the respective Ca^2+^ uptake experiments were immunoblotted with HA antibody, using β-actin as loading control. A representative immunoblot is shown. **e** Averaged Fura-2 ratio in arbitrary units (a.u.) of HEK293 cells expressing rabbit TRPV5 wild type (rbV5 WT, *red*; *n* = 69), human TRPV5 wild type (hV5 WT, *green*; *n* = 101), rabbit TRPV5 R707Q (rbV5 R707Q, *dark blue*; *n* = 57), and human TRPV5 Q706R (hV5 Q706R, *light blue*; *n* = 59) upon forskolin (FSK) stimulation at *t* = 60 s indicated by the *arrow*. The total cell number is obtained in three independent experiments. **f** Statistical analysis of Fura-2 ratio at the start (*t* = 0 s) and end of the experiment (*t* = 300 s). *p < 0.05 compared to *t* = 0 (basal start condition). **g** CaM binding assay of HEK293 cells transfected with human wild type TRPV5 and the indicated mutants. Samples were analyzed by immunoblotting with HA antibody. The CaM fraction represents the TRPV5 bound to the CaM agarose beads (*top panel*) and input demonstrates TRPV5 expression in total cell lysates (*bottom panel*). Representative immunoblot of three independent experiments is depicted

TRPV5 channel function was measured with a ^45^Ca^2+^ uptake assay in HEK293 cells that were transiently transfected with either wild type rbTRPV5 (rbV5 WT) or hTRPV5 (hV5 WT). Both rbTRPV5 and hTRPV5 were expressed as functional ion channels with substantial ^45^Ca^2+^ uptake (Fig. 1a). Ruthenium red (RR) was used as a blocker of TRPV5 to define the TRPV5-mediated ^45^Ca^2+^ uptake. The total lysate expression of hTRPV5 was slightly increased compared to rbTRPV5 (Fig. 1b). Expression levels of hTRPV5 and rbTRPV5 channels at the plasma membrane were assessed by cell surface biotinylation. Both hTRPV5 and rbTRPV5 were present in the biotinylated fractions, and demonstrated similar abundance at the cell surface when corrected for the input fraction (Fig. 1c). Of note, the immunoblot protein band pattern of hTRPV5 was different from rbTRPV5, which is likely due to glycosylaton differences (data not shown). Incubation with forskolin (FSK), a compound known to increase cAMP levels, stimulated rbTRPV5 function [6], but did not enhance hTRPV5-mediated uptake (Fig. 1a). In addition, the forskolin response on the intracellular Ca^2+^ concentration ([Ca^2+^]_i_) was assessed by Fura-2 Ca^2+^ imaging. Figure 1d demonstrates the Fura-2 ratio over time in HEK293 cells expressing mock, rbTRPV5 or hTRPV5. Addition of forskolin at *t* = 60 s resulted in a significant increase in rbTRPV5, but no response was observed in hTRPV5 (Fig. 1d). In order to extend these findings to PTH-mediated signaling, cells were co-transfected with the PTH receptor PTH1R, and incubated with the biologically active fragment of PTH, PTH(1–31). This is known to result in receptor activation, leading to downstream cAMP accumulation and PKA activation [6]. Incubation with PTH(1–31) did not enhance hTRPV5 activity, while rbTRPV5 was stimulated in line with reported data [6] (Fig. 2a, b). Of note, treatment with forskolin or PTH did not alter protein expression for either rbTRPV5 or hTRPV5 (Figs. 1b and 2c, d).

### Characterization of hTRPV5 Q706R and rbTRPV5 R707Q mutant channels

**Fig. 3:**
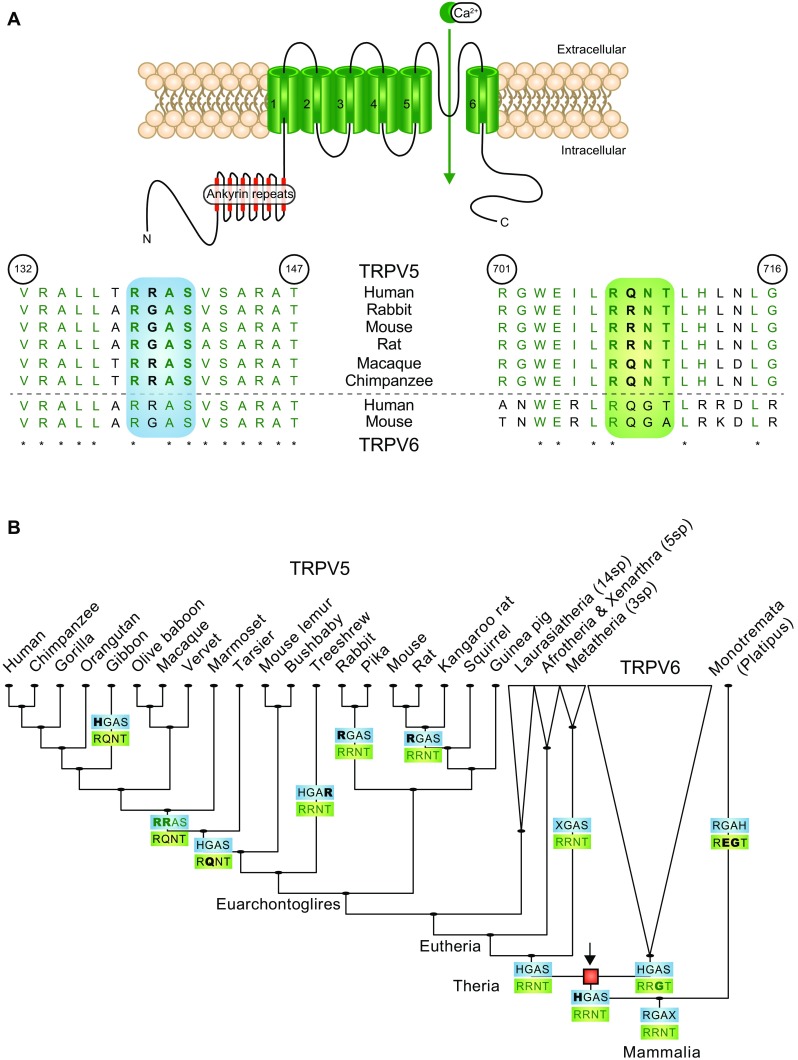
Ancestral sequence reconstruction of PKA phosphorylation motifs in mammalian TRPV5 orthologues. **a** Schematic overview of the TRPV5 monomeric subunit comprising six transmembrane segments and intracellular amino (N) and carboxy termini (C). A sequence alignment of amino acids 132–147 and 701–716 (human TRPV5) is depicted below in which different species sequences were compared using pBLAST. Highlighted in *green* is PKA consensus motif 1, RR/QxT (*right*). Highlighted in *blue* is PKA consensus motif 2, RR/GxS (*left*). Sequences are *green* upon conservation (also indicated with *asterisk*). **b** The amino-terminal PKA motif 2 (RR/GxS, *blue box*) and carboxy-terminal PKA motif 1 (RR/QxT, *green box*) are depicted for each point in evolution when one or both motifs changed based on parsimony-based ancestral sequence reconstruction. The red square marks (*arrow*) the point of duplication of TRPV5 and TRPV6. Motifs are colored *green* when matching the canonical RRxT/S motif. Non-matching motifs are depicted in *black*. The changed residues at each evolutionary event are noted by *bold font*. The carboxy-terminal PKA motif in rabbit appears to be the ancestral state, while the amino-terminal PKA motif in human has resulted from successive modifications to the motifs. TRPV5 and TRPV6 likely resulted from duplication in the ancestor of therian mammals

In rbTRPV5, the RRxT sequence has been established as the key phosphorylation motif for PTH-mediated PKA signaling, but this motif is not completely conserved among species [6]. The hTRPV5 sequence holds RQxT (Fig. 3a). To further determine the involvement of the RQxT/RRxT motif change in hTRPV5 channel function, mutant channels of these motifs were generated to mimic the opposite species, thereby creating hTRPV5 Q706R and rbTRPV5 R707Q. Subsequently, sensitivity to PTH was assessed with ^45^Ca^2+^ uptake assays. Remarkably, rbTRPV5 R707Q was no longer stimulated by PTH, while hTRPV5 Q706R was significantly stimulated by PTH incubation (Fig. 2a, b). Likewise, Fura-2 analysis demonstrated an increase in [Ca^2+^]_i_ upon addition of forskolin in hTRPV5 Q706R, while the forskolin response was abrogated in rbTRPV5 R707Q (Fig. 2e). Statistical analysis of the Fura-2 ratio at *t* = 0 s (−FSK) and *t* = 300 s (+FSK) showed a significant enhancement in wild type rbTRPV5 and hTRPV5 Q706R (Fig. 2f). In contrast, the Fura-2 ratio of wild type hTRPV5 and rbTRPV5 R707Q was not different between the unstimulated (−FSK) and forskolin-stimulated conditions (+FSK) (Fig. 2f). Of note, the wild type and mutant channels were expressed at equal levels (Fig. 2c, d).

Since PKA phosphorylation at T709 was shown to disrupt CaM binding, which involves R706 in rbTRPV5 (RRxT) [6], a CaM binding assay was used to examine the role of this motif in hTRPV5. Lysates from HEK293 cells expressing wild type hTRPV5, hTRPV5 Q706R, or rbTRPV5 R707Q were incubated with CaM agarose beads to demonstrate binding of full length hTRPV5 to CaM in the presence of Ca^2+^ (Fig. 2g). A condition in which cells were lysed in the presence of 5 mM EGTA was used as negative control since Ca^2+^ binding to CaM is essential for its interaction to other proteins. No differences in CaM binding were observed between the conditions tested (Fig. 2g, upper panel).

### Evolutionary conservation of the PKA phosphorylation motif

To provide more insight into the hTRPV5 RQxT sequence, the evolutionary development of the motif differences was examined by composing an ancestral sequence reconstruction of TRPV5 orthologues with pre-aligned sequences. This demonstrated that the RRxT motif has changed to an RQxT motif in higher primates and humans (Fig. 3a, b). Interestingly, the conversion of the carboxy-terminal RRxT motif into RQxT occurs together with an amino-terminal motif change of RGxS (rbTRPV5) into RRxS (hTRPV5) (Fig. 3b). This stretch was previously reported as another putative PKA consensus site by computational analysis [6]. Since the serine in the amino-terminal RGxS sequence (S142 rbTRPV5) was not shown to be involved in PTH-mediated PKA signaling in rbTRPV5, we were interested to understand whether the divergence in amino- and carboxy-terminal motifs holds functional differences for TRPV5 in humans.

### Characterization of hTRPV5 and rbTRPV5 phospho-mutant channels

**Fig. 4:**
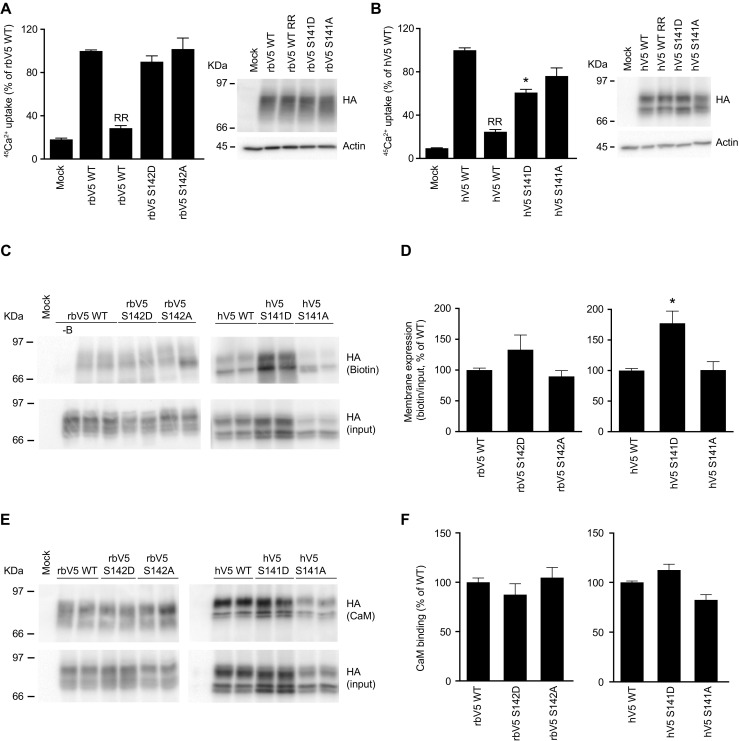
Characterization of amino-terminal phosphorylation mutants. **a**, **b** ^45^Ca^2+^ uptake assay of HEK293 cells transfected rabbit wild type TRPV5 (rbV5 WT) or the indicated mutants (S142D, S142A; **a**), and human wild type TRPV5 (hV5 WT) or the indicated mutants (S141D, S141A; **b**). Data is presented as percentage of rbV5 WT and hV5 WT, respectively, in mean ± SEM (*n* = 9, from three independent experiments). *Asterisk* indicates *p* < 0.05 compared to rbV5 WT and hV5 WT respectively. Ruthenium red (RR) is used to define TRPV5-mediated uptake. Cell lysates of the respective Ca^2+^ uptake experiments were immunoblotted with HA antibody, using β-actin as loading control. A representative immunoblot is shown in the right panels. **c** Cell surface biotinylation of HEK293 cells transfected with human (hV5 WT) or rabbit (rbV5 WT) wild type TRPV5. Samples were analyzed by immunoblotting with HA antibody. The biotin fraction represents the TRPV5 present at the plasma membrane (*top panel*) and input demonstrates TRPV5 expression in total cell lysates (*middle panel*). Representative immunoblot of three independent experiments is depicted. Control without added biotin is indicated as *-B*. **d** Quantification of the cell surface biotinylation experiments represents the relative membrane expression compared to input, and depicted as percentage of WT. *Asterisk* indicates *p* < 0.05 compared to hV5 WT. **e** CaM binding assay of HEK293 cells transfected with either rabbit wild type (rbV5 WT) or human wild type TRPV5 (hV5 WT) and the indicated mutants. Samples were analyzed by immunoblotting with HA antibody. The CaM fraction represents the TRPV5 bound to the CaM agarose beads (*top panel*) and input demonstrates TRPV5 expression in total cell lysates (*bottom panel*). Representative immunoblot of three independent experiments is depicted. **f** Quantification of the immunoblots is depicted as percentage of WT, which represents the relative CaM binding compared to input

Based on this evolutionary change, we investigated the role of hTRPV5 S141 phosphorylation. To this end, HEK293 cells, expressing the constitutively phosphorylated (SD) and non-phosphorylated (SA) mutants, were subjected to ^45^Ca^2+^ uptake assays. Interestingly, hTRPV5 S141D demonstrated a significantly reduced uptake compared to wild type hTRPV5 (Fig. 4b). The corresponding rbTRPV5 S142D and S142A mutants functioned similarly as wild type rbTRPV5 (Fig. 4a). Protein expression levels were not different between the conditions tested (Fig. 4a, b; right panels). Next, the abundance of the mutants at the plasma membrane was examined with cell surface biotinylation. Figure 4c shows a representative immunoblot of three independent experiments that were quantified (Fig. 4d). There were no significant differences between wild type and mutant rbTRPV5, but there was a significantly increased plasma membrane expression of hTRPV5 S141D compared to wild type hTRPV5 (Fig. 4d). Since the carboxy-terminal RRxT consensus motif, involved in CaM binding, has changed in hTRPV5, and there are indications that CaM may also bind the amino-terminal region of TRPV5 [17, 31], the effect of S141 phosphorylation on CaM binding was examined. Lysates of HEK293 cells expressing wild type TRPV5 and phosphorylation mutants were used for incubation with CaM agarose beads (Fig. 4e). Quantification of three independent binding assays resulted in no significant differences in CaM binding for the S141A/D mutants (Fig. 4f).

## Discussion

In the present study, rbTRPV5 and hTRPV5 were compared with respect to PTH-mediated PKA phosphorylation. Although both are present at the plasma membrane as functional highly Ca^2+^ selective channels, there are remarkable functional differences. While rbTRPV5 function is significantly stimulated upon PTH treatment, in a similar fashion as previously reported [6], our study showed that hTRPV5 is not stimulated by PTH or its downstream effector PKA. Our data indicates that the carboxy-terminal PKA consensus phosphorylation motif change (rbRRxT vs hRQxT) might explain the observed differences as hTRPV5 Q706R can be stimulated by PTH, while the R707Q mutation abrogates stimulation of rbTRPV5. Interestingly, ancestral sequence analysis demonstrated that this motif change coincided with conversion of a putative PKA motif at the amino terminus (RGxS > RRxS). In contrast to rbTRPV5, the constitutively phosphorylated mutant in hTRPV5 (S141D) exhibited decreased channel activity and demonstrated an enriched abundance at the plasma membrane.

The Ca^2+^ protective mechanism of long-term PTH treatment has been well established [24], but there is no information on the short-term molecular effects of PTH in humans. To our best knowledge, this is the first study demonstrating that humans might have lost or amended this non-genomic PTH signaling response on Ca^2+^ reabsorption in the kidney. In broader context, we essentially show that hTRPV5 cannot be stimulated by cAMP elevation and subsequent PKA activation, which is a common intracellular signaling event upon activation of various G-protein-coupled hormone receptors [26]. Therefore, it is likely that additional signaling mechanisms are controlling TRPV5 function in humans. Another example of species-specific signaling has previously been reported by Chamoux et al. [4]. Their study demonstrated that the RANKL-induced Ca^2+^ influx via phospholipase C activation is absent in human osteoclast in contrast to what has been observed in rodents [4]. They suggest that this is due to interspecies differences in the activation of TRP channels in response to RANKL. Because the large family of TRP channels can be activated upon various signals, including ligands and intracellular pathways, they are likely to be subjected to species-specific signaling.

The finding that hTRPV5 Q706R only showed partial stimulation suggests that factors beyond the RQxT/RRxT motif govern sensitivity to PTH. A search for additional putative PKA motifs in hTRPV5 outlined a sequence difference at residues 139–142 of hTRPV5, resulting in RRxS (conserved in Simian species) compared to RGxS in mouse, rat, and rabbit. Reconstruction of a phylogenetic tree demonstrated the occurrence of the carboxy- and amino-terminal motif changes at higher primates, suggesting an adaptation in the regulation of TRPV5 function. Our study shows that phosphorylation of S141 results in diminished function of hTRPV5, while the respective S142D mutation does not affect rbTRPV5. In addition, we demonstrated that hTRPV5 S141D is significantly more abundant at the plasma membrane, suggesting an effect of this mutation on the functional level. Future studies should confirm whether S141 can be phosphorylated in vivo and how channel function is affected.

It has previously been reported that CaM binds the amino terminus of rbTRPV5 (residues 133–154 and 310–330) [17]. Moreover, Tudpor et al. demonstrated that activation of protease-activated receptor-1 (PAR-1) by plasmin resulted in PKC phosphorylation at S144 in rbTRPV5, thereby inhibiting channel function. Using nuclear magnetic resonance spectroscopy (NMR), they showed that CaM binding is affected by phosphorylation of S144. While this site is in close proximity to S142 (S141 in hTRPV5), we did not observe significant differences in CaM binding upon phosphorylation. This could be due to either structural difference in S144 and S142 phosphorylation, or to the fact that full length TRPV5 is used for our CaM binding assay instead of amino-terminal peptides in the NMR study.

In addition to PAR1-mediated PKC phosphorylation of rbTRPV5 S144, it has been demonstrated that PKC can phosphorylate rbTRPV5 at S299 and S654, which is involved in channel membrane abundance via caveolae-mediated endocytosis [3, 10]. Importantly, the binding of PTH to the PTH1R was shown to activate the PLC pathway, leading to subsequent PKC activation [8, 29]. It was found that PTH-mediated stimulation of rbTRPV5 could be abolished by mutating S299/S654 [3]. These phosphorylation sites were also reported to be involved in activation of rbTRPV5 upon either stimulation of the bradykinin 2 receptor or the calcium-sensing receptor [10, 30]. PKC may also be involved in the regulation of hTRPV5 channel function. Hence, the direct effects of PTH-mediated PKC phosphorylation on the cell surface expression and the single channel activity of hTRPV5 are an important topic for further study. It is likely that the sequence differences between rbTRPV5 and hTRPV5 hold the answer, and it would be interesting to determine if the RQxT and RRxS motifs, present in hTRPV5, are able to serve as a site for PKA and/or PKC phosphorylation in a more physiological setting.

Thus, our study provides the first steps in the elucidation of the complex role of PTH-mediated PKA phosphorylation in the regulation of hTRPV5. Our data indicate interspecies differences in the regulation of hTRPV5 and rbTRPV5 that could change our present model of the physiological role of PTH in Ca^2+^ reabsorption. Specifically in humans, alternative hormone signaling pathways may be involved in renal Ca^2+^ handling, which would have implications for our current understanding of Ca^2+^ homeostasis in both health and disease.
